# Determination of the Optimal Training Principle and Input Variables in Artificial Neural Network Model for the Biweekly Chlorophyll-*a* Prediction: A Case Study of the Yuqiao Reservoir, China

**DOI:** 10.1371/journal.pone.0119082

**Published:** 2015-03-13

**Authors:** Yu Liu, Du-Gang Xi, Zhao-Liang Li

**Affiliations:** 1 Institute of Geographic Sciences and Natural Resources Research, Chinese Academy of Sciences, Beijing, China; 2 Institute of Remote Sensing and Digital Earth, Chinese Academy of Sciences, Beijing, China; 3 The PLA Information Engineering University, Zhengzhou, China; 4 Naval Institute of Hydrographic Surveying and Charting, Tianjin, China; 5 Key Laboratory of Agri-informatics, Ministry of Agriculture / Institute of Agricultural Resources and Regional Planning, Chinese Academy of Agricultural Sciences, Beijing, China; 6 ICube, UdS, CNRS, 300 boulevard Sebastien Brant, CS 10413, 67412 Illkirch, France; University of Connecticut, UNITED STATES

## Abstract

Predicting the levels of chlorophyll-*a* (Chl-*a*) is a vital component of water quality management, which ensures that urban drinking water is safe from harmful algal blooms. This study developed a model to predict Chl-*a* levels in the Yuqiao Reservoir (Tianjin, China) biweekly using water quality and meteorological data from 1999-2012. First, six artificial neural networks (ANNs) and two non-ANN methods (principal component analysis and the support vector regression model) were compared to determine the appropriate training principle. Subsequently, three predictors with different input variables were developed to examine the feasibility of incorporating meteorological factors into Chl-*a* prediction, which usually only uses water quality data. Finally, a sensitivity analysis was performed to examine how the Chl-*a* predictor reacts to changes in input variables. The results were as follows: first, ANN is a powerful predictive alternative to the traditional modeling techniques used for Chl-*a* prediction. The back program (BP) model yields slightly better results than all other ANNs, with the normalized mean square error (NMSE), the correlation coefficient (Corr), and the Nash-Sutcliffe coefficient of efficiency (NSE) at 0.003 mg/l, 0.880 and 0.754, respectively, in the testing period. Second, the incorporation of meteorological data greatly improved Chl-*a* prediction compared to models solely using water quality factors or meteorological data; the correlation coefficient increased from 0.574-0.686 to 0.880 when meteorological data were included. Finally, the Chl-*a* predictor is more sensitive to air pressure and pH compared to other water quality and meteorological variables.

## Introduction

Chlorophyll-*a* (Chl-*a*) is commonly used as an indicator of the abundance of phytoplankton and the population levels of primary productivity in the lakes and reservoirs that provide most of the drinking water for dozens of large and medium cities in China. Predicting the levels of Chl-*a* is a vital part of water quality management to ensure that urban drinking water is safe from harmful algal blooms.

Chl-*a* levels in lakes and reservoirs have been modeled for over 40 years [1], [2], and several statistical and process-based physical models have been developed using analysis of phytoplankton. Two of the most commonly used statistical predictors are linear regression models [3], [4] and principal component analysis [5], [6], [7]. These methods are simple but often do not yield reliable results, and sometimes even produce significant errors due to poor statistical stability and the use of linear equations. With improved understanding of aquatic ecosystem processes and advanced computing capabilities, physical models are now used to address water quality problems [8], [9], [10]. Although these models can describe variations in Chl-*a* levels based on the mechanism, they are not well suited for most Chinese lakes and reservoirs they require a significant amount of field data.

Artificial neural networks (ANNs), which imitate the basic characteristics of the human brain such as self-adaptability, self-organization and error tolerance, are able to map non-linear relationships among the variables that are typical of aquatic ecosystems [11]. Since their first application for the prediction of algal blooms from water quality databases of the Saidenbach Reservoir in Germany [12], ANNs have been widely applied to study Chl-*a*. Some examples of their application include prediction of algal blooms in Lake Kasumigaura in Japan [13], forecasting the incidence of *cyanobacteria* in the Murray River in Australia [14], estimation of the Chl-*a* levels in three water bodies in Turkey [15], analysis of algal bloom dynamics in the coastal waters of Hong Kong [16], elucidation of phytoplankton dynamics in the Nakdong River in Korea [17], prediction of the Chl-*a* levels in the Nanzui water area of Dongting Lake in China [18], and modeling of Chl-*a* levels during spring algal blooms in the Xiangxi Bay of the Three Gorges Reservoir in China [19]. These studies revealed that ANNs outperform traditional statistical models in modeling non-linear behavior and are more flexible than physical models because they require less detailed knowledge of the aquatic ecosystem. However, none of these studies encountered difficulties specific to modeling of the Yuqiao Reservoir, which has extensive submerged aquatic plants in addition to problems common to most reservoirs, such as abundant blue algae, limited data, highly variable water levels, and complex physical and chemical processes. Shallow water and appropriate nutrition conditions in the Yuqiao Reservoir have led to extensive growth of submerged aquatic plants.

Furthermore, although it is important to select the proper training method to improve prediction, few studies have systematically analyzed the performance of different ANNs in predicting Chl-*a* levels. Finally, almost all these studies used only water quality data as inputs, whereas meteorological factors that greatly affect the growth and accumulation of algae were rarely considered. Therefore, this study developed an accurate biweekly Chl-*a* predictor for the Yuqiao Reservoir by selecting appropriate training methods based on comparison of several ANN and non-ANN methods and by determining the appropriate model inputs including meteorological factors.

## Study Area and Data

### 1. Study area

**Fig 1:**
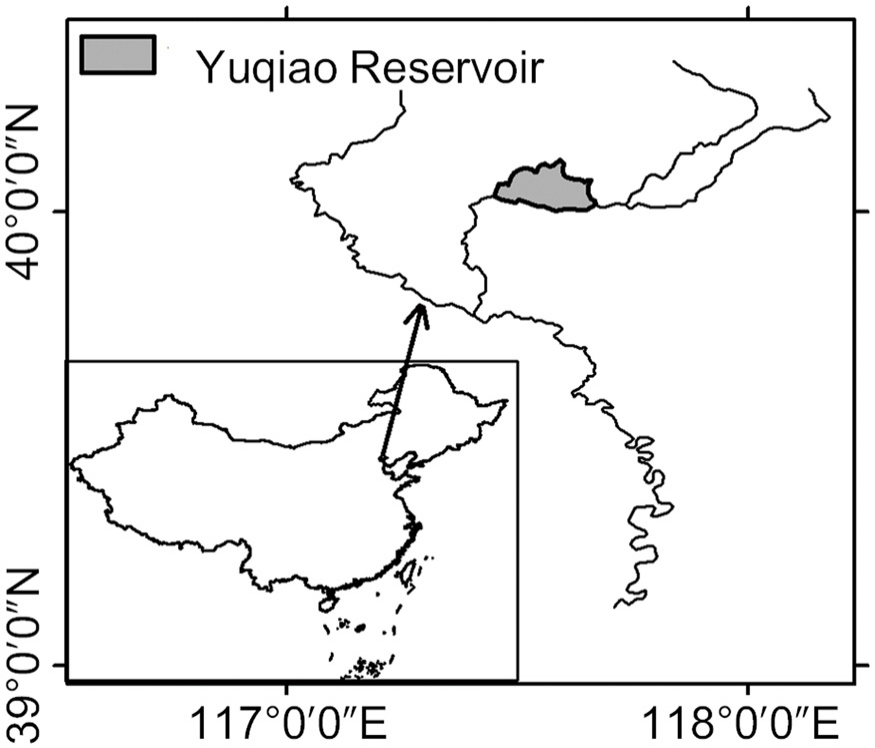
A map of the Yuqiao Reservoir in Tianjin, China.

The Yuqiao Reservoir (Fig. 1) is located downstream of the Haihe River Basin in northern China. It is the largest reservoir and the only source of drinking water for Tianjin, the third largest city in China with a population of 2.92×10^7^ in 2010. The reservoir was built in 1959 and used as a regulating reservoir during the diversion project from Luanhe to Tianjian in 1983. The reservoir surface area is 86.8 km^2^, and its volume and average depth at normal water level are 0.42×10^9^ m^3^ and 4.6 m, respectively. The mean annual precipitation and air temperature of the basin are 750 mm and 11.5°C, respectively.

The ecosystem of the Yuqiao Reservoir has undergone significant changes over the past few decades because of the natural evolution of biological species, changes in water diversion patterns, accelerated eutrophication of water quality, and substantial reduction of runoff resulting from climate change and human activities. The dominant species of submerged vegetation has changed from *Potamogeton maackianus* to *Potamogeton crispus*, and the biomass of *Potamogeton crispus* in late May increased from 4.8×10^7^ kg in 1988 to 1.19×10^8^ kg in 2009, whereas the distribution area of this species increased from 34.85% to 60.84%, according to remote-sensing estimates made using the Huanjing-1A/B satellite. The safety of the water supply of the Yuqiao Reservoir is now threatened by excessive growth of *Potamogeton crispus* in spring and algal outbreaks in summer. *Potamogeton crispus* is a submerged aquatic plant that purifies water by absorbing excess nutrients and competing for resources with *cyanobacteria* during its high growth period from April to mid-May. Furthermore, *Potamogeton crispus* promotes the growth of *cyanobacteria* by accelerating nutrient release during its explosive death and decay during late May and early June.

### 2. Data

(1) Samples. In this study, water quality and meteorological data from 1999–2012 were collected (S1 Data). The water quality data comprise 20 parameters including water temperature (Tw), pH, conductivity (Cond), transparency (Tran), chloride (CL), hardness (Hard), ammonia nitrogen (NH_4_-N), nitrate-nitrogen (NO_3_-N), nitrite-nitrogen (NO_2_-N), total nitrogen (TN), dissolved oxygen (DO), permanganate index (PI), biochemical oxygen demand (BOD), total phosphorus (TP), phosphate (PS), total solids (TS), suspended solids (SPS), soluble solids (SLS), salinity (SAL) and Chl-*a*. These data were collected by the Yuqiao Reservoir Administrative Bureau from the center of the reservoir (117°30′30.24″E and 40°02′35.02″N) approximately every two weeks in the summer and monthly in other seasons.

Meteorological data were obtained at the Tianjin site (117° 04′ E and 39° 05′ N) daily by the National Weather Service Information Center. These data include 11 parameters: mean air pressure (P), maximum air pressure (Pmax), minimum air pressure (Pmin), mean air temperature (Ta), maximum air temperature (Tamax), minimum air temperature (Tamin), precipitation (PCP), average wind speed (WS), maximum wind speed (WSmax), sunshine duration (SD), and total radiation (R).

(2) Features and variables. Feature extraction and determination of variables are important for any pattern recognition task, especially for Chl-*a* prediction, which involves complicated processes and variations. Excessive inputs may result in inefficient Chl-*a* prediction, whereas limited inputs may fail to describe the relationship between the influential variables and Chl-*a* levels.

To prepare features and variables for model inputs, we first interpolated the field water quality data into biweekly sets using a linear method and then processed the meteorological data to match the water quality data. The predicted day was set as Day_0_, and the current day was set as Day_15_. Therefore, the average value of the water quality and meteorological data of the preceding days 15–165 were processed into biweekly intervals. Second, considering the absence of field data for days 0–15 relative to the predicted day, we supplemented these data with the 10-year (2000–2009)-average water quality and meteorological variables of the corresponding period. Therefore, a total of 372 variables ((11 meteorological data + 20 water quality variables) × 12) were prepared (Table 1).

**Table 1:** Correlation coefficients of Chlorophyll-a (Chl-*a*) with water quality and Chl-*a* with meteorological variables.

Variables*	Day_0_ **	Day_15_ ***	Day_30_	Day_45_	Day_60_	Day_75_	Day_90_	Day_105_	Day_120_	Day_135_	Day_150_	Day_165_
Tw	0.28	0.33	0.58	0.58	0.59	0.57	0.55	0.51	0.47	0.42	0.36	0.3
pH	0.18	0.2	0.23	0.24	0.26	0.28	0.29	0.3	0.29	0.27	0.24	0.19
Cond	0.00	0.02	-0.05	-0.1	-0.15	-0.18	-0.2	-0.21	-0.22	-0.22	-0.23	-0.25
Tran	0.46	0.49	0.49	0.48	0.48	0.45	0.43	0.4	0.32	0.26	0.2	0.17
CL	0.14	0.19	0.17	0.15	0.13	0.1	0.09	0.06	0.04	0.01	-0.02	-0.06
Hard	-0.32	-0.33	-0.42	-0.44	-0.48	-0.5	-0.49	-0.47	-0.45	-0.41	-0.38	-0.34
NH_4_-N	0.05	0.09	0.02	-0.03	-0.1	-0.18	-0.23	-0.29	-0.35	-0.39	-0.42	-0.44
NO_3_-N	-0.55	-0.56	-0.55	-0.52	-0.49	-0.46	-0.43	-0.39	-0.36	-0.32	-0.28	-0.24
NO_2_-N	-0.07	-0.14	-0.16	-0.16	-0.17	-0.2	-0.24	-0.3	-0.36	-0.41	-0.46	-0.49
TN	-0.45	-0.42	-0.45	-0.44	-0.43	-0.42	-0.4	-0.38	-0.36	-0.32	-0.29	-0.26
DO	-0.4	-0.47	-0.51	-0.52	-0.51	-0.49	-0.45	-0.39	-0.31	-0.22	-0.14	-0.06
PI	0.59	0.57	0.57	0.59	0.57	0.53	0.48	0.42	0.35	0.28	0.21	0.13
BOD	0.11	0.03	0.01	0.02	0	-0.03	-0.06	-0.05	-0.06	-0.05	-0.04	-0.03
TP	0.54	0.46	0.53	0.56	0.56	0.54	0.52	0.46	0.39	0.33	0.27	0.19
PS	-0.04	0	-0.08	-0.11	-0.11	-0.13	-0.14	-0.15	-0.15	-0.16	-0.17	-0.17
TS	-0.07	-0.15	-0.15	-0.16	-0.19	-0.19	-0.17	-0.17	-0.17	-0.16	-0.18	-0.22
SPS	0.47	0.4	0.45	0.41	0.39	0.39	0.36	0.29	0.24	0.19	0.14	0.09
SLS	-0.15	-0.15	-0.15	-0.17	-0.18	-0.18	-0.18	-0.2	-0.2	-0.21	-0.22	-0.22
SAL	-0.24	-0.27	-0.28	-0.29	-0.3	-0.3	-0.28	-0.27	-0.26	-0.25	-0.25	-0.26
Chl-*a*	1.00	0.71	0.69	0.69	0.66	0.6	0.54	0.48	0.41	0.35	0.29	0.23
P	0.31	0.36	0.41	0.46	0.51	0.55	0.53	0.5	0.48	0.46	0.45	0.43
Pmax	0.28	0.34	0.4	0.45	0.5	0.55	0.52	0.51	0.48	0.47	0.47	0.46
Pmin	0.29	0.35	0.41	0.46	0.51	0.55	0.53	0.5	0.48	0.48	0.47	0.47
Ta	0.45	0.46	0.47	0.47	0.48	0.49	0.51	0.52	0.51	0.51	0.49	0.48
Tamax	0.41	0.42	0.43	0.43	0.45	0.46	0.48	0.49	0.48	0.48	0.46	0.45
Tamin	0.42	0.43	0.44	0.44	0.45	0.46	0.48	0.49	0.48	0.48	0.46	0.45
PCP	0.42	0.44	0.46	0.48	0.48	0.47	0.45	0.44	0.42	0.41	0.39	0.38
WS	0.29	0.36	0.36	0.38	0.37	0.29	0.28	0.27	0.26	0.26	0.25	0.25
WSmax	0.28	0.35	0.36	0.38	0.37	0.29	0.28	0.26	0.25	0.25	0.24	0.24
SD	0.23	0.25	0.3	0.31	0.3	0.28	0.26	0.24	0.24	0.23	0.23	0.22
R	0.21	0.25	0.31	0.34	0.32	0.29	0.26	0.23	0.22	0.22	0.21	0.21

Note:

* Tw, water temperature; Cond, conductivity; Tran, transparency; CL, chloride; Hard, hardness; NH_4_-N, ammonia nitrogen; NO_3_-N, nitrate-nitrogen; NO_2_-N, nitrite-nitrogen; TN, total nitrogen; DO, dissolved oxygen; PI, permanganate index; BOD, biochemical oxygen demand; TP, total phosphorus; PS, phosphate; TS, total solids; SPS, suspended solids; SLS, soluble solids; SAL, salinity; P, daily mean air pressure; Pmax, maximum air pressure; Pmin, minimum air pressure; Ta, average air temperature; Tamax, maximum air temperature; Tamin, minimum air temperature; PCP, precipitation; WS, average wind speed; WSmax, maximum wind speed; SD, sunshine duration; R, total radiation.

** Day_0_, data of the predicted day;

*** Day_15_, Day_30_…Day_165_, the average data of the previous 15, 30…165 days.

To reduce the dimensionality of the input data and to determine the appropriate model inputs, a threshold was applied to the correlation coefficient (Table 1). Variables whose correlation coefficient with Chl-*a* was over 0.5 were considered relatively important and selected as inputs. Therefore, a total of 27 variables of 6 water quality features (6 Tw variables: Tw_30_, Tw_45_, Tw_60_, Tw_75_, Tw_90_, and Tw_105_; 3 DO variables: DO_30_, DO_45_, and DO_60_; 3 PI variables: PI_30_, PI_45_, and PI_60_; 5 TP variables: TP_30_, TP_45_, TP_60_, TP_75_, and TP_90_; 5 NO_3_-N variables: Nia_0_, Nia_15_, Nia_30_, Nia_45_, and Nia_60_; 5 Chl-*a* variables: Chla_0_, Chla_15_, Chla_30_, Chla_45_, and Chla_60_) and 16 variables of 4 meteorological features (4 P variables: P_60_, P_75_, P_90_, and P_105_; 4 Pmax variables: Pmax_60_, Pmax_75_, Pmax_90_, and Pmax_105_; 4 Pmin variables: Pmin_60_, Pmin_75_, Pmin_90_, and Pmin_105_; 4 Ta variables: Ta_90_, Ta_105_, Ta_120_, and Ta_135_) were selected.

Based on field experience, meteorological variables such as WS, SD and R were added as inputs because of their close relationship to Chl-*a* despite their low correlation coefficients (<0.5), which result from the typical non-linear relationship between variables and Chl-*a*. Meteorological variables whose correlation coefficient with Chl-*a* was over 0.3 were also selected as inputs. Therefore, 14 meteorological variables (5 WS variables: WS_0_, WS_15_, WS_30_, WS_45_, and WS_60_; 5 SD variables: SD_0_, SD_15_, SD_30_, SD_45_, and SD_60_; 4 R variables: R_30_, R_45_, R_60_, and R_75_) were selected.

pH was also selected as an input despite a low correlation coefficient, similar to the WS meteorological variables.

Considering the similarity of air pressure variables, 3 air pressure features (P, Pmax and Pmin) were reduced to one (P), and 8 air pressure variables were excluded (4 Pmax variables: Pmax_60_, Pmax_75_, Pmax_90_, and Pmax_105_; 4 Pmin variables: Pmin_60_, Pmin_75_, Pmin_90_, and Pmin_105_).

Precipitation was not considered in this study despite relatively good correlation coefficients (0.38–0.48) because precipitation events were rare, and their values varied greatly, which might cause significant uncertainty in the prediction model.

Chla_0_ was excluded because by definition it was the predicted Chl-*a*, i.e., the 10-year-average Chl-*a*. The use of Chla_0_ might significantly influence the annual average Chl-*a* prediction model, making it less flexible to variations in water quality and meteorological conditions.

Therefore, a total of 49 variables of 12 features (27 variables of 7 water quality features and 22 variables of 5 meteorological features) were selected for this study (Table 2).

**Table 2:** Features and variables of the Chl-*a* prediction model.

Type	Features*	No. of variables	Variables**
Water quality	Tw	6	Tw_30_, Tw_45_, Tw_60,_ Tw_75_, Tw_90_, Tw_105_
	pH	1	pH_105_
	DO	3	DO_30_, DO_45_, DO_60_
	PI	3	PI_30_, PI_45_, PI_60_
	TP	5	TP_30_, TP_45_, TP_60_, TP_75_, TP_90_
	NO_3_-N	5	Nia_0_, Nia_15_, Nia_30_, Nia_45_, Nia_60_
	Chl-*a*	4	Chla_15_, Chla_30_, Chla_45_, Chla_60_
Meteorology	P	4	P_60_, P_75_, P_90_, P_105_
	Ta	4	Ta_90_, Ta_105_, Ta_120_, Ta_135_
	WS	5	WS_0_, WS_15,_ WS_30_, WS_45_, WS_60_
	SD	5	SD_0_, SD_15_, SD_30_, SD_45_, SD_60_
	R	4	R_30_, R_45_, R_60_, R_75_

Note:

* Features are the same as listed in Table 1.

** Average value of the variables over the indicated number of preceding days. For example, Tw_30_ represents the average water temperature of the preceding 30 days.

**Fig 2:**
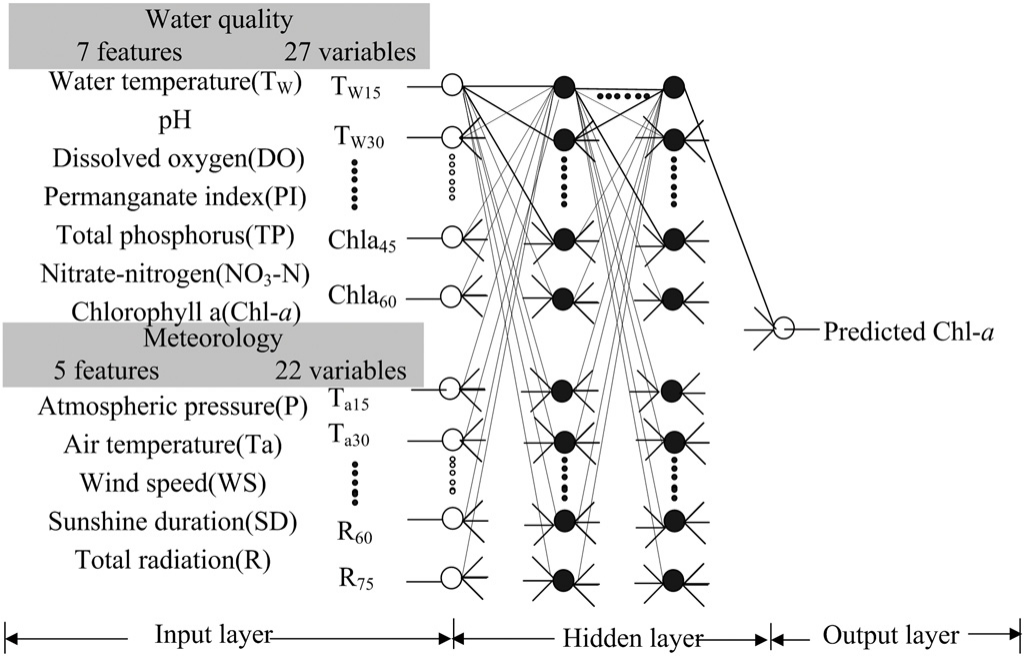
Configuration of the Chlorophyll-*a* (Chl-*a*) predictor using artificial neural networks (ANN). Left: input water quality and meteorological variables extracted by the correlation coefficient threshold method; middle: configuration of the predictor; right: predicted Chl-*a*. White circles on the left and right represent input and output neurons, respectively. Black circles represent neurons in the hidden layer. Lines around the circles indicate the data flow. A total of 49 variables of 12 features (27 variables of 7 water quality features and 22 variables of 5 meteorological features) were used.

(3) Configuration. Based on the above parameters, the Chl-*a* predictor was designed as shown in Fig. 2. The predictor comprises three parts: an input layer, an output layer and several hidden layers. Each layer contains several neurons. Each neuron receives inputs from neurons in the previous layers or from external sources and then converts the inputs either to an output signal or to another input signal for neurons in the next layer. The connections between neurons in successive layers were assigned weighted values, which represent the importance of that connection in the network.

## Methodology

This section introduces the strategy used to develop a Chl-*a* predictor, which considers factors including choice of an appropriate training method, determination of adequate model inputs, and identification of suitable network architecture and parameters.

### 1. Training method

To identify which model is best suited for the Chl-*a* predictor, the following six widely used ANNs were compared: Back Propagation (BP), Probabilistic Neural Network (PNN), Modular Neural Networks (MNN), Jordan-Elman network, Self-Organizing Map (SOM), and Co-Active Neuro-Fuzzy Inference System (CANFIS). BP is most likely the most widely used ANN and comprises a feed-forward multi-layer neural network in which connections can jump over one or more layers, and errors are propagated back to connections stemming from the input units. PNNs are nonlinear hybrid networks typically containing a single hidden layer of processing elements and use Gaussian transfer functions; all weights can be calculated analytically in these networks. MNNs combine the results from several parallel multilayer perceptrons. SOMs transform arbitrary dimensional inputs into a one- or two-dimensional discrete map considering topological constraints. CANFIS integrates adaptable fuzzy inputs with a modular neural network to rapidly and accurately approximate complex functions. These ANNs are described in detail in Liu et al. [20].

To examine the performance of ANNs, ANNs were compared to two typical traditional non-ANN methods: principal component analysis (PCA) and support vector machine (SVM). PCA is a widely used statistical method, which identifies relatively few “features” or components that as a whole represent the full object state. SVM geometrically separates the training set using a hyperplane or more complex surfaces if necessary; SVM is a new mathematical method, which is widely used in modeling ecosystems.

The ANN predictors were performed using the NeuroSolutions 6.31 (www.neurosolutions.com) software for the MATLAB neural network toolbox.

### 2. Model inputs

To examine the feasibility of including meteorological variables in the Chl-*a* predictor, which uses only water quality data, three models were constructed and analyzed using the following inputs: (a) only water quality factors (WQ) (27 variables of 7 features); (b) only meteorological factors (MF) (22 variables of 5 features); (c) both water quality and meteorological factors (WM) (49 variables of 12 features).

### 3. Evaluation indices

The performance of the Chl-*a* predictor was measured first by computational cost and then by precision. The first evaluation index was based on the training time required, and the second index was based on the normalized mean square error (NMSE), the correlation coefficient (Corr), and the Nash-Sutcliffe coefficient of efficiency (NSE). These evaluation indices are described below.

$$NMSE={\left(\frac{\sum_{i=1}^{N}{\left(y_{pi}\text{-}y_{i}\right)}^{2}}{N}\right)}^{1/2}$$ (1)

$$C\text{orr}=\frac{\sum_{i=1}^{N}\left(y_{pi}\;\times y_{i}\right)}{{\left(\sum_{i=1}^{N}{\left(y_{pi}\right)}^{2}\times \sum_{i=1}^{N}{\left(y_{i}\right)}^{2}\right)}^{1/2}}$$ (2)

$$NSE=1\text{-}\frac{\sum_{i=1}^{N}{\left(y_{pi}\text{-}y_{i}\right)}^{2}}{\sum_{i=1}^{N}{\left(y_{pi}\text{-}\bar{y}\right)}^{2}}$$ (3)

*y*_*pi*_ is the predicted Chl-*a* value at moment *i*, and *y*_*i*_ is the observed value; *N* is the number of days with interval of 15 days; $\bar{y}$is the average Chl-*a* value observed at all moments.

### 4. Model parameters

Selection of parameters such as the number of hidden layers, number of neurons, and learning rules, etc. was mainly based on the performance of NMSE, Corr, and NSE, which depended on the experience of the researcher and several tests. The Chl-*a* predictor was trained with maximum supervised epochs of 10000 times, and average MSE less than 0.01 were used as the termination constraint condition.

The learning momentum of the 6 ANN models was set as 0.7. A hyperbolic tangent function was used as the transfer function for axons (TanhAxons) as follows: f(*x*_*i*_,*w*_*i*_) = tanh(*x*_*i*_^*lin*^), where *x*_*i*_^*lin*^ = *βx*_*i*_ is the scaled and offset activity inherited from the linear axon. The learning momentums and TanhAxons were the same for SVM and PCA in the output layers.

Other parameters of the 8 Chl-*a* models with the same inputs are shown in Table 3. For the three models using different inputs, parameters were identical to the chosen model shown in Table 3 except for the number of neurons in the input and hidden layers. The number of neurons in the input layer for the models WQ, MF and WM were 27, 22, and 49, respectively, whereas the number of neurons in the first hidden layer were 30, 20 and 40, respectively, and in the second hidden layer, there were 25, 20 and 30 neurons, respectively.

**Table 3:** Parameters of the eight Chl-***a*** predictors.

Type of network*		Output layer		Hidden layer	
		Learning step	Number of hidden layers	Number of Neurons	Learning step
ANN	BP	0.1	2	40,30** ^(1)^	0.1
	MNN	0.1	1	45,30** ^(2)^	0.1
	Jordan-Elman *** ^(1)^	0.1	1	40	0.1
	PNN*** ^(2)^	1	1	60	1
	SOM*** ^(3)^	0.1	1	60	1
	CANFIS*** ^(4)^	0.1		50	0.1
Non-ANN	SVM	0.01			
	PCA)*** ^(5)^	0.1			

Note:

* BP, Back Propagation; MNN, Modular Neural Network; Jordan-Elman, Jordan-Elman network; PNN, Probabilistic Neural Network; SOM, Self-Organizing Map network; CANFIS, Co-Active Neuro-Fuzzy Inference System; SVM, the Support Vector Machine; PCA, Principal Component Analysis.

** Number of neurons: (1): Hidden layer 1: 40; Hidden layer 2: 30; (2): Upper processing elements (PEs) = 45; Low PEs = 30.

*** Some structural parameters of the models are (1) Time: 0.4; Integrator axon; (2): Cluster: 40; Competitive: conscience; Metric: Euclidean; (3) Rows: 4; columns: 10; Starting: 4; Final radius: 0; Neighborhood shape: Square Kononen Full; (4) Gamma axon memory; Depth in: 10; Trajectory: 50; (5) Learning rule: Sanger full; Principal 4. Further information on the parameters is available at http://www.neurosolutions.com/downloads/documentation.html.

### 5. Training and validation

Since 1983, when the Yuqiao Reservoir became the only source of drinking water for Tianjin city, the greatest changes in water quality, weather conditions and ecosystem in the reservoir occurred during 1999–2012. These changes occurred because of increased nutrient input, significant reduction of runoff, change in water diversion patterns, and natural evolution of ecosystems, which were closely related to climate change, urban water consumption, and newly built water conservancy projects in the upper reaches. The Chl-*a* level varied from 0.00–0.35 mg/l in 1999–2009 and from 0.00–0.28 mg/l in 2010–2012. The factors influencing the aquatic ecosystems were similar in 2010–2012 and 1999–2009, and there were no extreme weather conditions or changes in water utility patterns. Therefore, the prediction model was developed using data from 1999–2009 and tested using data from 2010–2012 because generally approximately 80% of the samples are used for training and the rest are used for testing while developing ecologic models.

Among the development data, seventy percent were randomly selected to train the model, and the remaining data were used for cross-validation. To avoid over-fitting the network, training was stopped if there was no improvement from the cross-validation process after 100 iterations. Weighted connection values were adjusted to minimize the RMSE between the desired and predicted outputs.

Because the training data spanned most cases of extreme conditions in the Yuqiao Reservoir since 1983, and the validation data were appropriate to test the performance of the proposed model, the Chl-*a* predictor should illustrate variations in Chl-*a* levels corresponding to changes in the ecosystem, weather conditions, and water diversion plans. Furthermore, the proposed model used a greater number of appropriate water quality factors and incorporated meteorological factors as inputs, whereas traditional predictors only use a limited number of water quality factors; therefore, compared to most traditional Chl-*a* predictors, the proposed model should adapt better to variations in weather conditions and water diversion patterns.

However, the performance of the model under new water diversion patterns and extreme weather conditions is unclear. This scope of this study did not include cases with limited water quality data and extreme conditions, which are low-probability events and occur randomly.

### 6. Sensitivity

To examine how the trained Chl-*a* predictor reacted to changes in each input, a sensitivity analysis was performed. Each input to the model was altered by 5%, 10% and 20%, and the corresponding change in output was calculated. For an input indicator to be considered sensitive, the corresponding output variation had to be greater than the input variation. A maximum input alteration of 20% was selected because some parameters such as pH and air pressure are relatively stable and vary by less than 20%.

## Results and Discussion

The performance of the Chl-*a* predictor was examined in three ways. First, 6 ANNs and 2 non-ANN predictors were compared to identify the appropriate model. Second, three ANN models with different input variables were developed to determine the feasibility of incorporating meteorological variables. Third, a sensitivity analysis was performed to examine how the trained network reacted to changes in each input.

### 1. Comparison of ANN and non-ANN predictors

Table 4 shows the results of the training, validation and testing of the eight Chl-*a* predictors. Except for the PNN method, all other methods required a training period that was less than 30 seconds. There were no time limits for most ANNs.

**Table 4:** Results of the eight Chl-*a* predictors with the same inputs.

Method*	Time (S)	Training			Validation			Testing		
		NMSE (mg/l)	Corr	NSE	NMSE (mg/l)	Corr	NSE	NMSE (mg/l)	Corr	NSE
BP	12.00	0.004	0.897	0.772	0.003	0.895	0.808	0.003	0.88	0.754
MNN	7.00	0.005	0.686	0.714	0.005	0.6	0.739	0.006	0.553	0.637
Jordan/El man	14.67	0.004	0.648	0.736	0.005	0.53	0.703	0.004	0.489	0.629
PNN	42.00	0.003	0.766	0.711	0.003	0.684	0.634	0.004	0.631	0.668
SOM	14.00	0.003	0.748	0.749	0.003	0.654	0.633	0.006	0.603	0.604
CANFIS	29.33	0.004	0.742	0.761	0.005	0.676	0.706	0.005	0.624	0.702
PCA	26.30	0.003	0.604	0.619	0.005	0.576	0.583	0.005	0.524	0.540
SVM	14.87	0.227	-0.050	0.49	0.341	-0.062	0.314	0.258	-0.057	0.491

Note:

* Methods are the same as shown in Table 2.

Except for the SVM method, the Corr between the observed and predicted Chl-*a* values of the ANNs was 0.524–0.880 in the testing period. This level of precision was consistent with similar studies on other water bodies, such as a correlation coefficient of 0.5–0.7 in the Putrajaya Lake of Malaysia [21] and 0.77 in the Nakdong River Basin of South Korea [17]. The performance of the ANN predictors was largely satisfactory considering the difficulties encountered in modeling the Yuqiao Reservoir, which contains extensive submerged aquatic plants in addition to the complex physical, chemical, and biological processes observed in other water bodies. Furthermore, the long-term series training data extending over 11 years (1999–2009) also contributed to the complexity of Chl-*a* prediction because the important factors governing Chl-*a* values changed significantly over time. For example, *Potamogeton crispus* became the dominant submerged aquatic plant with biomass and distribution areas that doubled every May over the past 30 years.

The training precision for the predictors was ranked in the following order based on the NMSE, Corr, and NSE evaluation indices: BP > MNN, CANFIS, SOM, Jordan/Elman, PNN > PCA > SVM. The results obtained during validation and testing were consistent with results obtained during training.

In this study, all 6 ANNs outperformed non-ANN methods. For example, the NSE of ANNs during testing was 0.604–0.754, whereas the NSE of the PCA and SVM methods was 0.540 and 0.491, respectively. The failure of the PCA and SVM methods may result from the complex nonlinear nature of the Yuqiao Reservoir ecosystem.

Among the ANNs, the BP method best predicted Chl-*a* levels with NMSE, Corr and NSE values of 0.003 mg/l, 0.880, and 0.754, respectively, during testing. However, there was no clear advantage of one ANN over others because all 6 ANN models yielded acceptable results.

The SVM method is not suitable for Chl-*a* prediction because Corr was < 0.1 and NSE was < 0.50 during the training, validation and testing periods; this is potentially because the SVM method treats multi-category problems as a series of binary problems and may thus fail to capture the high variability of the aquatic system in the Yuqiao Reservoir.

In conclusion, the performance of the eight predictors indicated that ANNs, especially when trained by the BP method, are a powerful alternative to traditional modeling techniques for Chl-*a* prediction.

### 2. Incorporation of meteorological variables

**Fig 3:**
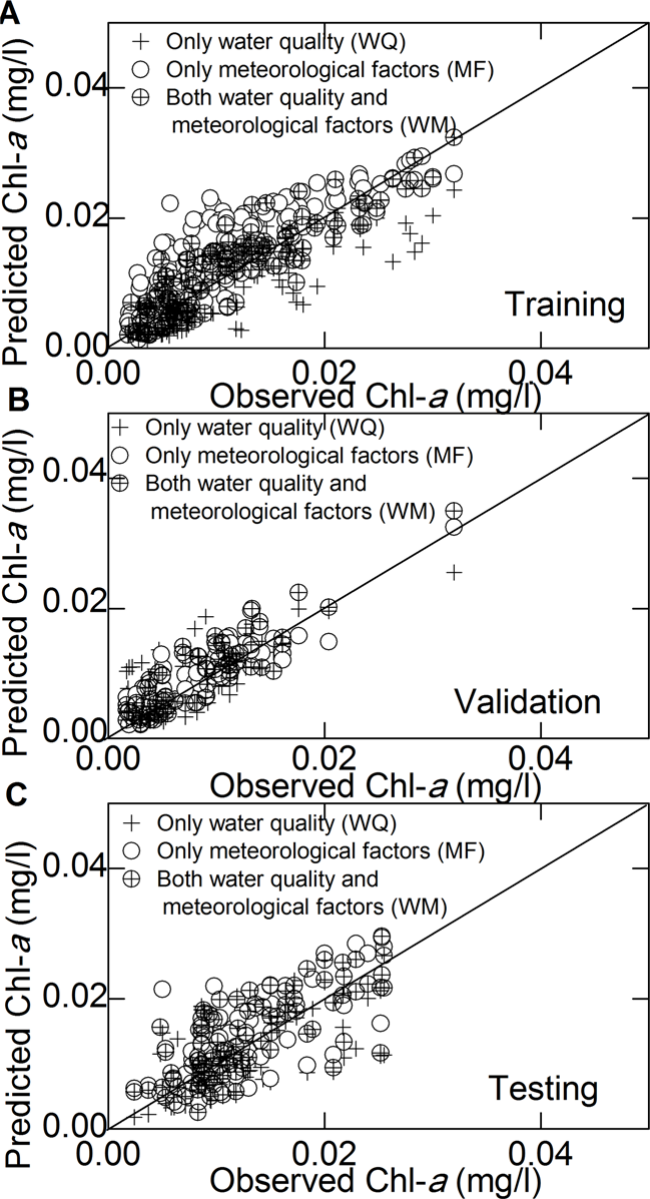
Scatter plots of the observed data vs. the model predictions using different inputs. (A) Chl-*a* prediction with different variables used as inputs for training; (B) is the same as (A) but for validation; (C) is the same as (A) but for testing.

Fig. 3 and Table 5 show the results of the three ANN models with different inputs of water quality and meteorological variables. The model with only meteorological factors (MF) as inputs always overestimated the concentration of Chl-*a*, whereas the model with only water quality variables (WQ) as inputs underestimated Chl-*a*, which was evident during the training period. Combining the water quality and meteorological variables (WF) improved the performance of the Chl-*a* predictor greatly by accurately detecting peak timing and magnitude. For example, the Corr of the WF model was 0.880, whereas the Corr of the WQ and MF models was only 0.574 and 0.686, respectively. The NSE of the WF model was 0.754, whereas the NSE of the WQ and MF models was 0.225 and 0.662, respectively.

**Table 5:** Results of three Chl-*a* predictors with different inputs

Evaluation indices	Training			Validation			Testing		
	WQ*	MF**	WF***	WQ	MF	WF	WQ	MF	WF
RMSE (mg/l)	0.005	0.008	0.005	0.003	0.003	0.003	0.004	0.005	0.003
Corr	0.710	0.844	0.897	0.735	0.884	0.895	0.574	0.686	0.880
NSE	0.395	0.679	0.772	0.361	0.674	0.808	0.225	0.662	0.754

Note:

*, **, and *** indicate models using only water quality factors, only meteorological factors, and both water quality and meteorological factors as inputs, respectively.

### 3. Sensitivity

**Fig 4:**
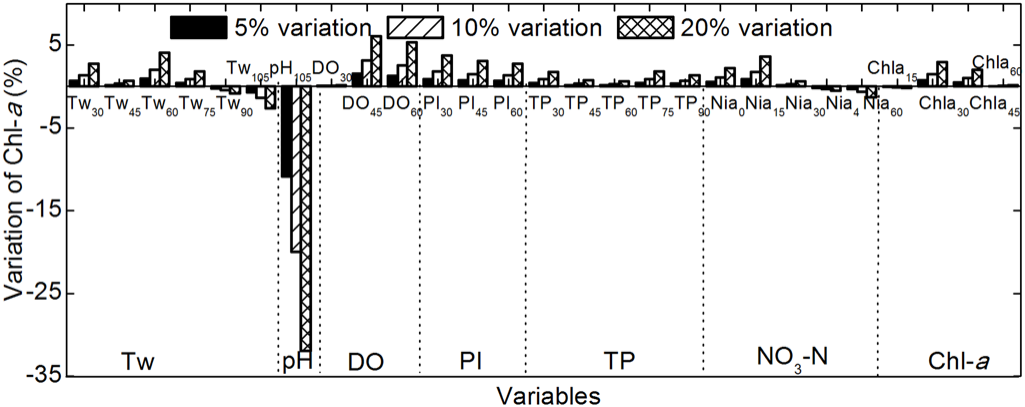
Sensitivity of the predictor to water quality variables. Bars indicate changes in Chl-*a* values caused by changes in the input variables, which were altered by 5%, 10% and 20%. Black, slash-filled, and cross line-filled bars indicate the change in Chl-*a* values caused by 5%, 10%, and 20% changes in input variables, respectively. Tw, water temperature; DO, dissolved oxygen; PI, permanganate index; TP, total phosphorus; NO_3_-N, nitrate-nitrogen.

The sensitivity of the Chl-*a* predictor to water quality variables is shown in Fig. 4. The sensitivity decreased in the following order: pH > DO, Tw, PI > NO_3_-N, TP and the prior Chl-*a*.

Tw has a short-term positive effect on the Chl-*a* concentration but a negative impact over longer durations. For example, the concentration of Chl-*a* increases with increasing water temperature in the preceding 30–75 days but reduces with increasing water temperature in the preceding 90–105 days. This may be because warm water promotes the growth of algae in the summer and *Potamogeton crispus* in the spring; excessive growth of *Potamogeton crispus* can inhibit the growth of algae by competing for nutrients and light.

Chl-*a* is very sensitive to pH variations, and the Chl-*a* concentration increases at twice the rate of pH change. This is potentially because a slight decrease in pH may significantly promote algal photosynthesis by increasing the dissolution of CO_2_ in water.

A higher Chl-*a* value generally implies a higher level of DO. To some extent, the level of DO can indicate how much oxygen is produced by phytoplankton.

PI and TP have similar effects on Chl-*a*, and Chl-*a* is relatively more sensitive to the permanganate index than to TP. This is because PI can indicate the abundance of phytoplankton, whereas TP influences Chl-*a* indirectly by promoting the growth of phytoplankton.

Chl-*a* has similar sensitivity to NO_3_-N and water temperature: Chl-*a* first increases then decreases with increasing NO_3_-N.

The Chl-*a* concentration is closely related to the Chl-*a* level during the preceding 15–60 days. This indicates that algal seeds significantly influence growth and Chl-*a* levels in the subsequent two months.

**Fig 5:**
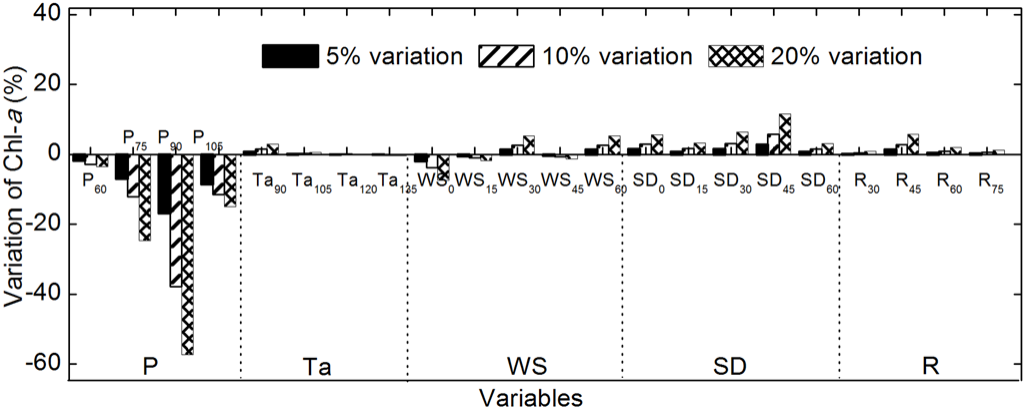
Sensitivity of the predictor to meteorological variables. Bars indicate changes as described for Fig. 4. P, daily average air pressure; Ta, average air temperature; WS, wind speed; SD, sunshine duration; R, total radiation.

The sensitivity of the Chl-*a* predictor to meteorological variables is shown in Fig. 5. The sensitivity decreased as follows: P > WS, SD, and R > Ta.

The Chl-*a* concentration increases rapidly as P decreases because low air pressure promotes floating and accumulation of algae on the water surface.

Chl-*a* is almost completely insensitive to changes in Ta; the Chl-*a* level varied by less than 5% when Ta was altered by 20%. This is because air temperature influences the aquatic system indirectly with water as a medium.

WS has a short-term negative effect and a long-term positive effect on Chl-*a* levels. This is because strong wind promotes the release of nutrients from the sediment, which promotes Chl-*a* increase in a relatively slow manner; however, strong wind rapidly inhibits the growth and accumulation of algal particles.

Longer SD and R periods result in increased Chl-*a* because they increase energy input to the aquatic ecosystem, which promotes photosynthesis.

Sensitivity to meteorological variables is meaningful for short-term forecasts and for long-term prevention of algal blooms. For example, consecutive days with low air pressure, slight wind speed and increasing SD and R in summer indicate a higher probability of algal bloom, which can help water quality management departments to implement advance countermeasures.

## Conclusion

To develop an appropriate biweekly Chl-*a* predictor for the Yuqiao Reservoir, this study first compared several Chl-*a* predictors trained using different methods and then examined the feasibility of incorporating meteorological factors for prediction. In addition, a sensitivity analysis was performed to examine how the Chl-*a* predictor reacted to changes in each input. The following observations were made:

(1) ANN is a powerful predictive alternative to traditional modeling techniques for Chl-*a* prediction with Corr values of 0.524–0.880 in the testing period. The BP model yields better results compared to other ANN models.
(2) Combining the water quality and meteorological data greatly improves the performance of the Chl-*a* predictor compared to models using water quality or meteorological data alone as inputs; the Corr values increased from 0.574–0.686 to 0.880 when both inputs were combined.
(3) Among the meteorological variables, Chl-*a* is most sensitive to air pressure, followed by wind velocity, sunshine duration, total radiation, and air temperature. Chl-*a* is more sensitive to changes in pH compared to other water quality variables such as DO, water temperature, NO_3_-N, TP and prior Chl-*a* values.

## Supporting Information

S1 Data (XLS)
